# Survival and predictors of mortality among children co-infected with tuberculosis and human immunodeficiency virus at University of Gondar Comprehensive Specialized Hospital, Northwest Ethiopia. A retrospective follow-up study

**DOI:** 10.1371/journal.pone.0197145

**Published:** 2018-05-22

**Authors:** Kendalem Asmare Atalell, Nigusie Birhan Tebeje, Daniale Tekelia Ekubagewargies

**Affiliations:** 1 Department of Pediatrics and Child Health Nursing, School of Nursing, College of Medicine and Health Sciences, University of Gondar, Gondar, Ethiopia; 2 School of Nursing, College of Medicine and Health Sciences, University of Gondar, Gondar, Ethiopia; The Ohio State University, UNITED STATES

## Abstract

**Background:**

Tuberculosis (TB) is the leading cause of death in Human immunodeficiency virus (HIV) infected children globally. The aims of this study were to determine the mortality rate and to identify the predictors of mortality among TB/HIV co-infected children at University of Gondar Comprehensive Specialized Hospital.

**Method:**

A retrospective follow-up study was conducted among TB/HIV co-infected children from February 2005 to March 2017. A Kaplan–Meier curve was used to estimate the median survival time. Bivariate and multivariable Cox proportional hazards models were fitted to identify the predictors of mortality.

**Results:**

A total of 271 TB/HIV co-infected children were included in the analysis. Of these, 38(14.02%) children were died during the follow-up period. This gives a total of 1167.67 child-years of observations. The overall mortality rate was 3.27(95%CI: 2.3–4.5) per 100 child-years. The independent predictors of time to death were age 1–5 years (as compared to age <1 year) (AHR = 0.3; 95%CI:0.09–0.98)), being anemic (AHR = 2.6; 95%CI:1.24–5.3), cotrimoxazole preventive therapy(CPT) non-users (AHR = 4.1; 95%CI:1.4–16.75), isoniazid preventive therapy(IPT) non-users (AHR = 2.95; 95%CI:1.16–7.5), having extra pulmonary tuberculosis(EPTB) (AHR = 2.43; 95%CI:1.1–5.3)) and fair or poor adherence to Anti-Retroviral Therapy (ART)(AHR = 3.5; 95%CI:1.7–7.5).

**Conclusion:**

Mortality rate among TB/HIV co-infected children was high at University of Gondar Comprehensive Specialized Hospital. Age, extra-pulmonary tuberculosis, anemia, adherence, CPT and IPT were the independent predictors of mortality.

## Background

Tuberculosis (TB) is the leading cause of death among HIV-infected children [1]. It is a major public health problem especially in low and middle-income countries [2–4]. According to the World Health Organization (WHO), in 2016, there were an estimated 10.4 million new cases of TB globally (equivalent to an incidence rate of 142 cases per 100,000 population) and 1.4 million deaths due to TB in 2015 [5]. Approximately, 1.2 million cases of TB occurred in HIV-positive people and 1.0 million cases occurred in children. The burden of TB/HIV co-infection is particularly high in sub-Saharan Africa including in Ethiopia [6].

Ethiopia has been classified as one of the 30 high TB and TB/HIV burden countries [5, 7]. The country is striving to reduce the magnitude of TB and HIV disease in line with the objectives of the sustainable development goal (SDG) [8]. However, the problem still remains high, particularly in children. TB is one of the top ten causes of death [9] and the most commonly reported opportunistic infection in children infected with HIV [10–13]. Even though there were advances in the implementation of prevention of mother to child transmission (PMTCT), and provision of isoniazid preventive therapy (IPT) in Ethiopia, TB is a major cause of hospital admission and death in HIV infected children [14]. The management of TB/HIV co-infection in children is very challenging especially in resource-limited settings such as Ethiopia because of the unavailability of appropriate formulations of drugs, a drug to drug interactions, pill burdens, drug side effects and poor adherence [15–17]. This resulted in high mortality rate among TB/HIV co-infected children.

As of 2015, a WHO report indicated that there were nearly 41,000 children were died due to TB/HIV co-infection. Of these, 34,000 were occurred in Africa [5]. The mortality rate of TB/HIV co-infection was varied in different settings and ranged from 11% to 36.5% [18, 19]. The cause of death among TB/HIV co-infected children are multi-factorial [20]. These include age, nutritional status, immunity status, hemoglobin level, use of CPT and IPT [21]. Thus, the aim of this study was to estimate the mortality rate and to identify the predictors of mortality among TB/HIV co-infected children at University of Gondar Comprehensive Specialized Hospital, Northwest Ethiopia.

## Method

### Study setting

The study was conducted at University of Gondar Comprehensive Specialized Hospital HIV care clinic. The hospital is found in Northwest Ethiopia and serves for more than 5 million people in North Gondar and neighboring zones. The HIV care service was established in January 2005. A total of 8581 adults and 1138 children were enrolled in HIV care until March 2017.

### Study design and participants

An institutional-based retrospective cohort study was conducted from February 2005 to March 2017. All TB/HIV co-infected children age less than 15 years who were enrolled in Pediatric HIV care Clinic at University of Gondar Comprehensive Specialized Hospital were eligible for this study.

### Data collection tools and procedures

Data were collected from medical records using data extraction checklist adapted from National HIV intake and follow-up forms. The checklist comprised socio-demographic, clinical and follow-up variables. The data were collected by four BSc. Nurses working at Pediatric HIV care clinic who had comprehensive HIV care training. The pre-test was conducted among 15 medical records to check the consistency of the data extraction checklist.

### Data analysis

Data were entered into EPI-info version 7 and then exported to STATA version 12 for analysis. WHO Anthro-Plus software was used to classify indices variables and to assess the nutritional status of the children. Descriptive statistics were carried out and summarized using tables and graphs. Mortality rate was calculated by dividing the number of children died during the follow-up period by the Child-Years of follow-up. Kaplan–Meier curve was used to estimate the median survival time. The Log-rank test was used to compare survival curves between the categories of the explanatory variables. A life table was used to estimate the probability of survival at a different time interval in the follow-up time.

Both bivariate and multivariable Cox proportional hazard model were used to identify the predictors of time to death of TB/HIV co-infected children. A bivariate Cox proportional hazard model was first fitted, and the variables significant at P-value <0.2 in the bivariate analysis were selected for the final multivariable Cox proportional hazard model. Then the final Cox proportional hazard model was fitted using backward stepwise selection. Variables having p-value less than 0.05 at 95% CI in the final multivariable Cox proportional hazards model were considered as significantly associated with the dependent variable. The necessary assumption of Cox proportional hazard model was checked by using Schoenfield residuals test.

### Ethical considerations

Ethical clearance was obtained from the Institutional Review Board of the University of Gondar. Permission letter was also obtained from University of Gondar Comprehensive Specialized Hospital’s management and HIV care clinics focal person to use the secondary data for the purpose of this study. Since we used secondary data, we did not get informed consents from each study participants.

## Results

### Socio-demographic characteristics

A total of 301 TB/HIV co-infected children’s medical records were reviewed. Of these, 30(9%) were excluded from the analysis due to missing of data. The remaining, 271 TB/HIV co-infected children were included in the analysis. The mean age of the study participants was 6.6(±3.5 SD) years. Nearly one-third 88 (32.47%) of the children were under 5 years and half of them 137(50.55%) were males (Table 1). The majorities 219 (80.81%) of the respondent were living in urban and 220 (81.18%) children were lives with their parents. Half 135 (49.82) of children’s caregiver were between the age group of 25 and 34 years with a median age of 30 (IQR (27–38)) years. Approximately, two third 176 (64.94%) of the children’s caregivers were HIV positive.

**Table 1:** Socio-demographic characteristics of TB/HIV co-infected children at University of Gondar Comprehensive Specialized Hospital, Northwest Ethiopia, from February 2005 to March 2017 (n = 271).

Characteristics		Total N (%)	Death N (%)	Censored N (%)
		N = 271	N = 38	N = 233
**Age (Years)**	<1	11(4.06)	5(1.85)	6(2.21)
	1–5	77(28.41)	8(2.95)	69(25.46)
	6–10	125(46.13)	19(7.01)	106(39.11)
	11–14	58(21.40)	6(2.21)	52(19.19)
**Sex**	Male	137(50.55)	19(7.01)	118(43.54)
	Female	134(49.45)	19(7.01)	115(42.44)
**Age of caregiver(Years)**	15–24	32(11.81)	5(1.85)	27((9.96)
	25–34	135(49.82)	21(7.75)	114(42.07)
	35–44	68(25.09)	7(2.58)	61(22.51)
	>44	36(13.28)	5(1.85)	31(11.44)
**Residence**	Urban	219(80.81)	28(10.33)	191(70.48)
	Rural	52(19.19)	10(3.69)	42(15.50)
**Family size**	< = 2	44(16.24)	10(3.69)	34(12.55)
	2–4	142(52.40)	18(6.64)	124(45.76)
	> = 5	85(31.37)	10(3.69)	75(27.68)
**Caregiver of the child**	Parents	220(81.18)	31(11.4)	189(69.74)
	Siblings	19(7.01)	1(0.37)	18(6.64)
	Grand-parents	23(8.49)	3(1.11)	20(7.38)
	Others	9(3.32)	3(1.11)	6(2.21)
**Child lives with**	Parents	247(91.14)	34(12.55)	213(78.60)
	Orphaned	11(4.06)	2(0.74)	9(3.32)
	Others	13(4.80)	2(0.74)	11(4.06)

### Clinical characteristics

A total of 237(87.45%) children had an advanced baseline WHO clinical stage (i.e. 3 and 4) (Table 2). More than one-third 95 (35.06%) of children had experienced with an initial regiment change during the follow-up period. Of these, 33(34.74%) were due to TB infections. Twenty-eight (10.33%) children had experienced with ART treatment failure. Of these 8(28.6%) children were initiated second-line ART.

**Table 2:** Clinical characteristics of TB/HIV co-infected children at University of Gondar Comprehensive Specialized Hospital Northwest Ethiopia, from February 2005 to March 20017 (n = 271).

Characteristics		Total N (%) N = 271	Death N (%) N = 38	Censored N (%) N = 233
**Baseline WHO stage**	I & II	34(12.55)	3(1.11)	31(11.40)
	III & IV	237(87.45)	35(12.90)	202(74.54)
**ART Eligibility criteria**	CD4+ cell	37(13.65)	4(1.48)	33(12.80)
	WHO stage	104(38.38)	11(4.06)	93(34.32)
	Both	125(46.13)	23(8.49)	102(37.32)
	Not recorded	5(1.85)	0(0)	5(1.85)
**Initial ART regiment based on NRTIs**	ABC-based	10(3.69)	5(1.85)	5(1.85)
	AZT-based	185(68.27)	20(7.38)	165(60.89)
	D4T-based	67(24.72)	12(4.43)	55(20.30)
	TDF-based	9(3.32)	1(0.37)	8(2.95)
**Initial ART regiments based on NNRTs**	EFV-based	102(37.64)	12(4.43)	90(33.21)
	NVP, PI and other based	169(62.36)	26(9.59)	143(52.77)
**Initial regiment change**	Yes	95(35.06)	13(4.80)	82(30.26)
	No	176(64.94)	25(9.23)	151(55.72)
**Reason for regiment change**	Side effect/toxicities	23(24.21)	6(6.32)	17(17.89)
	Treatment failure	2(2.11)	0(0)	2(2.11)
	TB	33(34.74)	5(5.26)	28(29.47)
	Stock out	37(38.95)	2(2.11)	35(36.84)
**Treatment failure**	Yes	28(10.33)	5(1.85)	23(8.49)
	No	243(89.67)	33(12.18)	210(77.49)
**Immunologic failure**	Yes	20(7.38)	4(1.48)	16(5.90)
	No	251(92.62)	34(12.55)	217(80.07)
**Virologic failure**	Yes	17(6.27)	3(1.11)	14(5.17)
	No	254(93.73)	35(12.92)	219(80.81)
**Clinical failure**	Yes	5(1.85)	3(1.11)	2(0.74)
	No	266(98.15)	35(12.92)	233(85.24)
**Baseline HIV associated Immunosuppression status**	Non-significant/Mild	87(32.10)	8(2.95)	79(29.15)
	Advanced	73(26.95)	8(2.95)	65(23.99)
	Sever	111(40.96)	22(8.12)	89(32.84)
**Isoniazid**	Yes	97(35.79)	6(2.21)	91(33.58)
	No	174(64.21)	32(11.81)	142(52.40)
**Hemoglobing/dl**	<10	48(17.70)	14(5.17)	34(12.50)
	> = 10	223(82.29)	24(8.86)	199(73.43)
**Co-trimoxazole preventive therapy**	Yes	236(87.08)	28(10.33)	208(76.75)
	No	35(12.92)	10(3.69)	25(9.23)
**Weight for age**	Normal	77(28.41)	12(4.43)	65(23.99)
	Underweight	194(71.59)	26(9.59)	168(61.99)
**Height for age**	Normal	110(40.59)	14(5.17)	96(35.42)
	Stunting	161(59.41)	24(8.86)	137(50.55)
**Adherence**	Good	231(85.24)	23(8.49)	208(76.75)
	Fair	27(9.96)	12(4.43)	15(5.53)
	Poor	13(4.80)	3(1.11)	10(3.69)
**Site of TB**	PTB	186(68.63)	13(4.80)	173(63.84)
	EPTB	85(31.37)	25(9.23)	60(22.14)
**Time at which TB is developed**	PRE ART	206(76.01)	25(9.23)	181(66.79)
	ART	65(23.99)	13(4.80)	52(19.19)

Forty-eight (17.7%) children were anemic at baseline with a median Hgb level of 12 (IQR; 10.6–13). Regarding prophylaxis use, 236(87.45%) of the respondents were on co-trimoxazole Preventive Therapy (CPT) and ninety-seven (35.79%) were on isoniazid preventive therapy (IPT). At TB diagnosis, stunting and underweight were 161(59.41%) and 194(71.59%) respectively.

### Mortality rate

Two hundred seventy-one children were followed for different periods (1 month to 12 years) that gives a total of 1167.67 Child-Years of observation. The median follow-up period was 4(IQR; 1.9–6.5) years. From a total of 271 children who were included in the analysis, 38(14.02%) new deaths were observed, 186 (68.6%) were alive at the end of the follow-up, 22 (8.1%) were transfer out to other treatment centres, and 25 (9.2%) were lost to follow-up. Thus, the overall mortality rate was 3.27 (95%CI: 2.43–4.52) per 100 Child-Years. Among children who died during the follow-up period, half (50%) of them were males and 23 (60.5%) died within the first year of follow-up (Table 3). Twenty-five children had extra-pulmonary or/and disseminated tuberculosis.

**Table 3:** Mortality rate stratified by socio-demographic and clinical characteristics of TB/HIV co-infected children at University of Gondar Comprehensive Specialized Hospital, Northwest Ethiopia, from February 2005 to March 2017.

Characteristics		Total N (%)	PY	Death N (%)	IDR
**Age (years)**	<1	11(4.06)	56.10	5(1.85)	8.92
	> = 1	260(95.94)	1105.20	33(12.18)	2.99
**Residence**	Urban	219(80.81)	1015.25	28(10.33)	2.76
	Rural	52(19.19)	152.40	10(3.69)	6.56
**Child lives with**	Parents	247(91.14)	1094.58	34(12.55)	3.10
	Orphaned	11(4.06)	36.00	2(0.74)	5.56
	Others	13(4.80)	37.10	2(0.74)	5.40
**Baseline WHO stage**	I & II	34(12.55)	114.25	3(1.11)	2.63
	III & IV	237(87.45)	1047.00	35(12.9)	3.34
**Baseline immunity status**	Non-significant/Mild	87(32.10)	337.83	8(2.95)	2.37
	Advanced/ severe	184(67.90)	823.42	30(11.07)	3.64
**Initial ART regiment based on NRTIs**	ABC-based	10(3.69)	20.17	5(1.85)	24.81
	AZT-based	185(68.27)	828.33	20(7.38)	2.40
	D4T-based	67(24.72)	298.17	12(4.43)	4.00
	TDF-based	9(3.32)	20.99	1(0.37)	4.81
**Treatment failure**	Yes	28(10.33)	149.58	5(1.85)	3.34
	No	243(89.67)	1018.10	33(12.18)	3.24
**Clinical failure**	Yes	5(1.85)	12.67	3(1.11)	23.70
	No	266(98.15)	1155.00	35(12.92)	3.00
**Isoniazid**	Yes	97(35.79)	542.00	6(2.21)	1.11
	No	174(64.21)	625.66	32(11.81)	5.11
**Hemoglobin g/dl**	<10	48(17.71)	167.58	14(5.17)	8.41
	> = 10	223(82.29)	1000.10	24(8.86)	2.40
**CPT**	Yes	236(87.08)	1079.58	28(10.33)	2.60
	No	35(12.92)	88.10	10(3.69)	11.40
**Adherence**	Good	231(85.24)	1037.92	23(8.49)	2.22
	Fair/poor	40(14.76)	123.33	15(5.54)	12.20
**Site of TB**	PTB	186(68.63)	801.67	13(4.80)	1.62
	EPTB	85(31.37)	365.999	25(9.23)	6.83
**Follow-up years**	<1	44(16.24)	15.33	23(8.49)	150.00
	1–5	116(42.8)	345.92	9(3.32)	2.60
	>5	111(40.96)	800.00	6(2.21)	0.75

**Fig 1:**
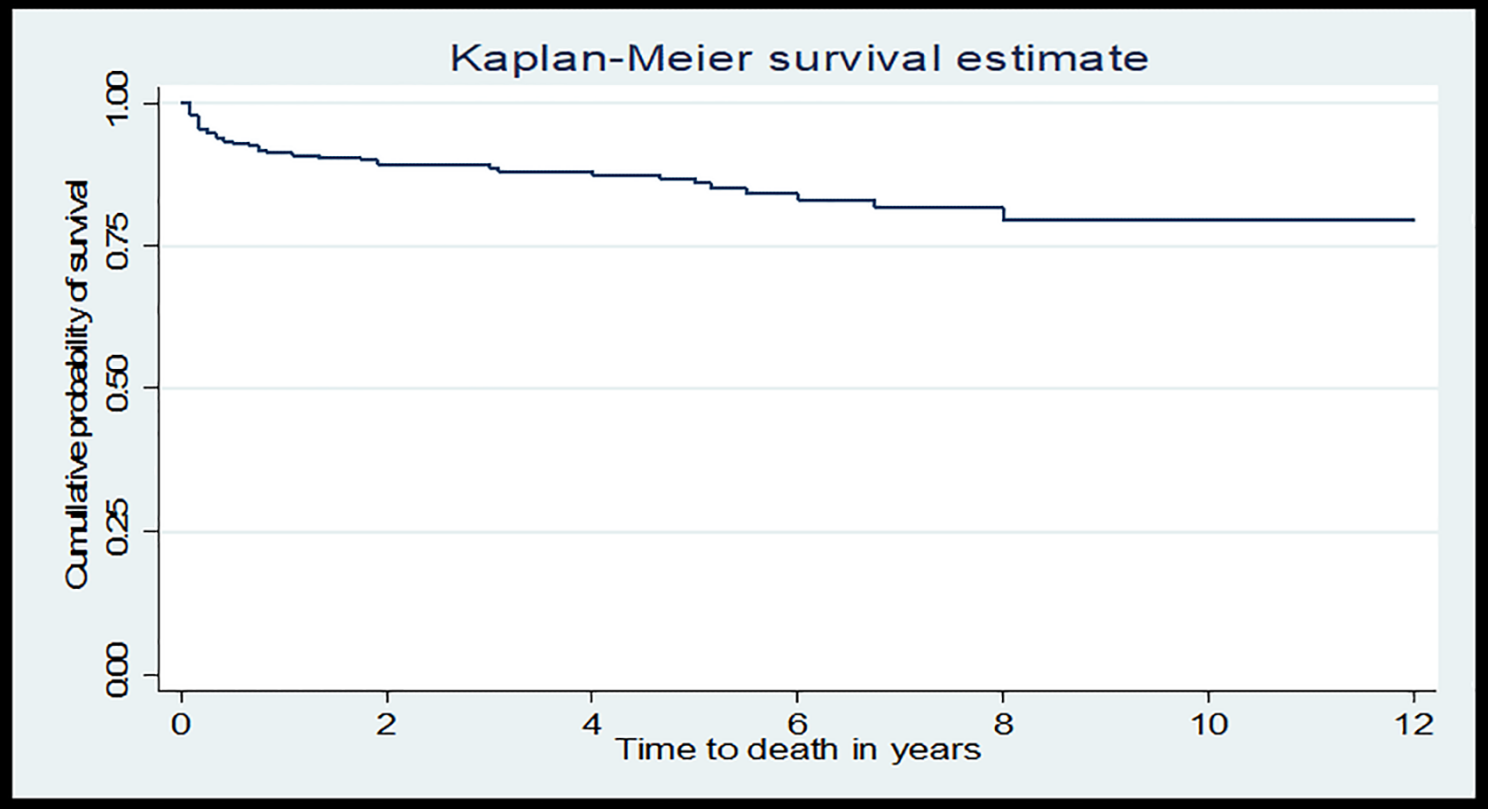
Kaplan-Meier curve of survival proportion for TB/HIV co-infected children at University of Gondar Comprehensive Specialized Hospital, Northwest Ethiopia, from February 2005 to March 2017.

The cumulative probability of survival at the end of 1 year was 91.2%, at the end of 3 years was 88.6%, at the end of 5 years was 85.8%, and at the end of 12 years was 79.4% respectively (Fig 1).

### Predictors of mortality

In the bivariate Cox Proportional Hazard model age, Hemoglobin level, co-trimoxazole preventive therapy (CPT), isoniazid prophylaxis (IPT), site of tuberculosis (TB) infections, severe immunosuppression and adherence to ART were statistically significant (Table 4). However, in multivariable Cox-Proportional Hazard model age, CPT, IPT, site of TB infection, adherence to ARV drugs and hemoglobin level remained statistically significant.

**Table 4:** Predictors of time to death of TB/HIV co-infected children at University of Gondar Comprehensive Specialized Hospital, Northwest Ethiopia, from February 2005 to March 2017 (n = 271).

Characteristics		Death	Censored	CHR(95%CI)	AHR(95%CI)
**Age**	<1year	5	6	1.00	1.00
	1–5 years	8	69	0.25 (0.08–0.77)*	0.3(0.09–0.98)**
	5–10	19	106	0.37(0.14–1.00)	0.7(0.22–2.3)
	10–14	6	52	0.29(0.09–0.96)	0.33(0.084–1.33)
**Mother HIV status**	Positive	22	160	1.00	1.00
	Negative/Unknown	16	73	1.77(0.93–3.37)	1.14(0.26–5)
**Address**	Urban	28	191	1.00	1.00
	Rural	10	42	1.99(0.96–4.11)	1.58(0.696–3.6)
**HIV status of caregiver**	Positive	22	154	1.00	1.00
	Negative /Unknown	16	79	1.57(0.82–2.997)	1.69(0.38–7.5)
**Immunity status at TB diagnosis**	N.S & mild	8	86	1.00	1.00
	Advanced IS	7	59	1.17(0.42–3.23)*	1.3(0.44–3.94)
	Sever IS	23	88	2.32(1.04–5.199)*	2.45(0.97–6.2)**
**Anemia status at TB diagnosis**	Anemic	14	34	3.1(1.6–6)*	2.6(1.24–5.3)**
	Non-anemic	24	199	1.00	1.00
**CPT**	Yes	28	208	1.00	1.00
	No	10	25	3.51(1.68–7.31)*	4.1(1.76–9.7)**
**IPT**	Yes	6	91	1.00	1.00
	No	32	142	3.87(1.6–9.28)*	2.95(1.16–7.45)**
**Site of TB**	PTB	13	173		
	EPTB	25	60	4.43(2.26–8.67)*	2.43(1.1–5.3)**
**Adherence**	Good	23	208	1.00	1.00
	Fair/poor	15	25	4.86(2.5–9.3)*	3.57(1.7–7.5)**
**Time of TB occurrence**	Pre ART	25	181	1.00	1.00
	ART	13	52	1.68(0.86–3.29)	1.46(0.68–3.15)

N.S = non-significant, I.S = immunosuppression.

* = variables significant in the baviriable at p-value less than 0.05 at 95%CI.

** = variables significant in multivariable at p-value less than 0.*05 at 95%CI*. *Anemic*: <10 mg/dl; non-anemic: ≥10 mg/dl.

According to the analysis, children whose age group 1–5 years were less likely to die as compared to children with age less than one year (AHR = 0.3; 95%CI: 0.09–0.98). Anemic Children were 2.6 times at higher risk of death as compared to non-anemic children (AHR = 2.6; 95%CI: 1.24–5.3). Similarly, children with extra-pulmonary or/and disseminated tuberculosis were 2.43 times at higher risk of death as compared children with pulmonary tuberculosis (AHR = 2.43; 95%CI: 1.1–5.3). Children who did not use CPT were 4.1 times at higher risk of death than children who used CPT (AHR = 4.1; 95%CI = 1.76–9.7). Similarly, IPT non-users were 2.95 times at higher risk of death as compared with IPT users (AHR = 2.95; 95%CI = 1.16–7.45).

A child with fair or poor adherence to ART drugs was 3.57 times at higher risk of death than a child with good adherence to ART drugs.

## Discussion

This is the first published study that has presented the mortality rate and predictors of mortality from a cohort of TB and HIV co-infected children in Northwest Ethiopia. The overall mortality rate was 3.27 (95%CI: 2.4–4.5) per 100Child-Year of follow-up. This result is consistent with mortality rate reported in systematic review and meta-analysis [22], and in other studies conducted in high TB and TB/HIV burden countries such as South Africa [23, 19, 24], and Indian [25]. However, our finding is higher than a study conducted in Nigeria (1.4 per 100 Child-Year follow-ups) [26].

The highest mortality rate (8.92/100CY) was observed in the first year of follow-up. The peak mortality rate in the first year might be associated with the progression of the sub-clinical disease, which remains undetected during enrollment and progresses rapidly. Late arrival at health care means late diagnosis which is one predictor of death among TB/HIV co-infected children supported by a study conducted in South Africa [27]. The other fact could be Immune reconstitution inflammatory syndrome (IRIS) which is common within 6 months of ART initiation. The result also showed that a high number of children were started ART within the first year of follow-up which increases the probability of IRIS occurrence [28].The other possible reason for increased TB/HIV co-infected children survival with duration of follow-up could be the result of the progressive increase in CD4 cell counts which builds the immune system and this may again decrease the viral load across time, finally, increase the survival rate.

The cumulative survival rate in this study was 79.4% (95%CI: 71% -85.6%) which is in line with a retrospective cohort study conducted in Nigeria 73% [29]. The survival rate in our study at 1, 2 and 3 years were 91.2%, 89.1%, and 88.6% respectively, which were similar with a cohort study in Thailand 96.1%, 94% and 87.7% at 1, 2 and 3years respectively among ART users, and much higher than 44.4%, 19.2%, and 9.3% among non-ART user group [20]. The discrepancy in those of pre-ART may be due to the effect of ART drugs. Anti-retroviral drugs are responsible for viral suppression, which increase the CD4 cell, finally, increase the survival of children and decrease the risk of death which is supported, by a study conducted in Malawi [30].

In our study, similar with other studies conducted in South Africa [23] and Nigeria [31], TB/HIV co-infected children with age less than one year were at higher risk of death than children with age 1–5 years. This is due to the fact that, children with age less than one year had an immature immune system, especially in TB/HIV co-infected children who have the tendency to develop the more severe disease, that leads to death. Anemic children were at higher risk of death than non-anemic children, which were similar to studies conducted in Tanzania [32], and Malawi [30]. This might be due to the effect of anemia on the oxygen intake capacity which had a synergistic effect with tuberculosis and HIV co-infections that increase the prognosis of the disease process which may end-up with death.

Co-trimoxazole preventive therapy non-users were four times at higher risk of death than CPT users. This may be due to the fact that CPT can prevent most of the opportunistic infections in HIV and TB co-infected children and, finally CPT use may reduce the mortality rate. Isoniazid Preventive Therapy (IPT) non-users were also at higher risk of death than IPT users. Similar findings were reported in South Africa [33] and Nigeria [31]. IPT prevents the reoccurrence of TB infections, severity and dissemination of TB. Our result showed that a child infected with extra-pulmonary or/and disseminated TB was at higher risk of death than a child infected with PTB.

We have also found that children who had fair or poor adherence to ART drugs had a higher risk of death than children who had good adherence to ART drugs. Similar findings were reported in other studies conducted in Indian [18] and Addis Ababa, Ethiopia [34]. Adhere to ART drugs will suppress viral replications and increase CD4 cells counts. This increases the survival of children and reduces mortality. On the other hand, children who had poor adherence to ART drugs will face several problems such as treatment failure, drug-resistant, the occurrence of OIs that could lead to death and poor outcomes. Thus, it would be important to provide treatment adherence counseling to the parents and caregivers of the child at the start of treatment and during the follow-up periods.

This study has some limitations. First, since this study was based on secondary data, some important variables were not available in the registers and therefore were not included in our study.

Second, those study subjects whose chart was not available in the ART clinic were not included in the study which may undermine the result if it is related to the study outcome. Finally, since we were unable to record the baseline socio-demographic and clinical characteristics for the incomplete and excluded records, we could not compare the study outcome between the excluded and included study subjects.

## Conclusion

Mortality rate was high among TB/HIV co-infected children at University of Gondar Comprehensive Specialized Hospital, Northwest Ethiopia. Age less than one year, having extra-pulmonary tuberculosis, being anemic, having fair or poor adherence, co-trimoxazole preventive therapy non-user, and isoniazid preventive therapy non-user were significant predictors of mortality among TB/HIV co-infected children.

## Supporting information

S1 File Excel support data for PLOS. (XLS)
